# German cranial reconstruction registry – a prospective multicenter cohort study: 883-day follow-up on the outcome and complications^☆^

**DOI:** 10.1016/j.bas.2025.104308

**Published:** 2025-07-02

**Authors:** Maximilian Bschorer, Henrik Giese, Julius Höhne, Karl Michael Schebesch, Christian Henker, Andreas Strauss, Christina Wolfert, Khaled Gaber, Aleksandrs Krigers, Ondra Petr, Vicki M. Butenschoen, Sandro M. Krieg, Klaus Christian Mende, Dirk Lindner, Jan Regelsberger, Dorothee Mielke, Thomas Sauvigny

**Affiliations:** a Department of Neurosurgery, University Medical Center Hamburg-Eppendorf, Hamburg, Germany; b Department of Neurosurgery, Hospital Center Braunschweig, Braunschweig, Germany; c Department of Neurosurgery, University Hospital Heidelberg, Heidelberg, Germany; d Department of Neurosurgery, Paracelsus Medical University Nuernberg, Nuernberg, Germany; e Department of Neurosurgery, University Hospital Rostock, Rostock, Germany; f Department of Orthopedic Surgery and Spinal Surgery, KMG Clinics Guestrow, Guestrow, Germany; g Department of Neurosurgery, Georg-August-University, Goettingen, Germany; h Department of Neurosurgery, University Hospital Augsburg, Augsburg, Germany; i Department of Neurosurgery, University Hospital Leipzig, Leipzig, Germany; j Department of Neurosurgery, Medical University of Innsbruck, Innsbruck, Austria; k Department of Neurosurgery, Klinikum Rechts der Isar, Technical University Munich, Munich, Germany; l Department of Neurosurgery, Friedrich-Ebert-hospital Neumuenster, Neumuenster, Germany; m Department of Neurosurgery, Diako Hospital Flensburg, Flensburg, Germany

**Keywords:** Cranioplasty, Decompressive craniectomy, Neurological outcome, Stroke, Traumatic brain injury, Glascow outcome score, Computer aided design

## Abstract

**Introduction:**

This international prospective multicenter cohort study investigates the long-term surgical complication rate and neurological outcomes in patients who underwent autologous or allogeneic cranioplasty (CP) after decompressive craniectomy (DC) for traumatic brain injury, stroke, aneurysmatic subarachnoid hemorrhage, and intracranial hemorrhage.

**Research question:**

This study investigated the predictors of long-term outcomes and surgical revision after cranioplasty.

**Materials and methods:**

Patients who underwent CP with a minimum follow-up of at least 12 months were included. Favorable long-term outcome (FLTO) was defined as a Glasgow Outcome Score (GOS) of 4 or 5 and a modified Rankin scale (mRS) score of <4. Univariate and multivariate analyses were performed.

**Results:**

A total of 200 patients with a median follow-up of 883.1 ± 520.5 days were included. Ninety-nine patients (50.0 %) had a FLTO, and the surgical revision rate was 25.0 % (n = 50). Thirty-eight percent (37.7 %) and 27.5 % of patients showed improvement in the mRS and GOS scores, respectively. Simultaneous implantation of a ventriculoperitoneal shunt (OR 6.114) and a time interval of <90 days between DC and CP (OR 2.189) predicted an increase in reoperation rates. The use of subcutaneous drains with suction predicted a lower rate of reoperation (OR .410). Diabetes mellitus (OR .221) and reoperations during the initial stay (OR .347) were negative predictors of FLTO. Implants imbued with antibiotics predicted a positive FLTO (OR 2.973).

**Discussion and conclusion:**

Suction drains were predicted to reduce reoperation rates. Simultaneous implantation of VPS and CP within 3 months of DC predicted an increased likelihood of surgical revision.

## Highlights

• The surgical revision rate after cranioplasty was 25 %.
• After cranioplasty, 37.7 % of patients showed improved neurological status.
• Suction drains were associated with a lower long-term surgical revision rate.
• Simultaneous ventriculoperitoneal shunt placement was associated with a higher long-term surgical revision rate.
• Cranioplasty within 90 days of decompressive craniectomy was associated with an increased surgical revision rate.

## 1 Introduction

Decompressive hemicraniectomy (DC) is performed to prevent an imminent loss of brain function due to refractory intracranial pressure after malignant infarction of the middle cerebral artery, diffuse traumatic brain injury (TBI), intracerebral hemorrhage (ICH), and aneurysmal subarachnoid hemorrhage (ASAH) (Vahedi et al., 2007; Cooper et al., 2011). Despite improved survival rates, patients often suffer from neurocognitive and neurological deficits for the rest of their lives and often have to undergo further neurosurgical procedures such cranioplasty (CP) and ventriculoperitoneal shunt (VPS) placement. (Cooper et al., 2011). Cranioplasty (CP) has become an important cornerstone in modern neurological rehabilitation because an increase in cerebral perfusion, normalization of cerebrospinal fluid (CSF) hydrodynamics, and intracranial pressure (ICP) can improve the long-term neurological outcomes of patients (Fodstad et al., 1984; Halani et al., 2017; Shahid et al., 2018). CP has been linked to enhanced patient safety by reestablishing a protective barrier that shields the brain parenchyma from external impacts. Additionally, it addresses the increased risk of falls due to neurological morbidity and contributes to improved cosmetic outcomes (Giese et al., 2021).

The German Cranial Reconstruction Registry (GCRR) is a prospective multicenter cohort study with the goal of analyzing postoperative neurological outcomes and the risk of complications after CP (Giese et al., 2015). The initial report on postoperative complications after CP reported a surgical revision rate of 9 % within the first 30 days after CP (Sauvigny et al., 2021). Other studies have also reported a high risk of complications associated with neurosurgical procedures, despite many surgeons deeming it a routine and technically unchallenging procedure (Zanaty et al., 2015). While the general morbidity of patients who undergo DC is a factor, surgical aspects such as large wounds and the head microbiome contribute to the unfavorable risk profile of this type of surgery (Chang et al., 2010).^1^

This poses an ethical dilemma. Patients with severe neurological impairments and other clinical risk factors are presented with the option of CP, a procedure that carries significant perioperative risks, with the hope of both cosmetic and neurocognitive improvements. While there have been multiple prospective multicenter studies on the 30-day outcomes after CP, only retrospective data have been published so far on the long-term results after CP(Sauvigny et al., 2021; Fountain et al., 2021). The timing of CP following DC has been a subject of considerable debate within the neurosurgical community (Vreeburg et al., 2024). In this study, we present the long-term neurological and surgical outcomes of the patients analyzed in this prospective cohort study and aim to provide both patients and healthcare providers with information that may guide future clinical practice.

## 2 Methods

The section for Neurotrauma and Intensive Care in Neurosurgery of the German Society for Neurosurgery initiated the GCRR as a procedure-specific prospective cohort study (Giese et al., 2015). All cases of CP after DC at the 16 participating centers in Germany and Austria were screened for inclusion in the study. A standardized questionnaire on CP was used to record patient-specific data, including risk factors, surgical details, materials used for CP, intraoperative and postoperative complications, and clinical follow-up (FU) at discharge. The case report forms (CRF) for CP and postoperative monitoring were specifically designed to evaluate and address the questions of this study **(Supplemental Digital Content** 1). These forms were used to record all complications from the day of discharge to readmission within 30 days after surgery and again during follow-up by physicians at participating centers (Sauvigny et al., 2021). Neurological status and medical comorbidities were assessed using two different outcome scales: the modified Rankin Scale (mRS) and Glasgow Outcome Score (GOS).

Local ethics committees approved this study for the participating hospitals. This study was conducted in accordance with the ethical standards of the Declaration of Helsinki. Written informed consent was obtained from the patient or the patient's authorized representative prior to inclusion. Pseudonymized data were collected centrally at the Department of Medical Biometry, Heidelberg, and transmitted to an electronic database. This trial was indexed in the German Clinical Trials Register (DRKSID no. DRKS00007931). The Universal Trial Number is U1111–1168–7425.

Only centers that reported on at least 50 % of patient cases with a minimum FU of at least 12 months were further analyzed to reduce bias. This aims to minimize selection bias due to loss of follow-up. In addition, only cases with valid data and a follow-up of at least 12 months were included in this analysis. The primary endpoint was defined as cranioplasty-associated reoperation (CPAR), which included any neurosurgical procedure after the initial CP, including VPS revision surgeries. The second primary endpoint was neurological outcome. A favorable long-term outcome (FLTO) is defined as a follow-up GOS greater than three and a mRS score less than four.

Statistical correlation of clinical data was performed using univariate analysis with the chi-square test, Mann-Whitney *U* test, or 2-sample *t*-test, depending on the distribution of the measurements. Cases of deviating statistical test results are indicated in the respective passages. Variables with significant p-values in the univariate analyses or clinical relevance were considered as potentially independent variables in the multivariate analysis. Odds ratios and 95 % confidence intervals were calculated using logistic regression models. The regression models for both CPAR and LFTO were adjusted for age, diagnosis, and sex to specifically target the surgical aspects of CP. Pairwise deletion was used for the missing data. All statistical analyses were performed using IBM SPSS Statistics version 28 (IBM Corp.) under the supervision of a statistician.

## 3 Results

### 3.1 Patients

Eight of the 16 participating centers fulfilled the inclusion criteria. In total, 529 cases were initially reviewed for inclusion in the registry between September 2015 and December 2019. Overall, 200 patients fulfilled the inclusion criteria for the long-term analysis. The median follow-up was 883.1 ± 520.5 days. The average age of patients at the time of CP was 49.5 ± 16.07 years. The average age of patients receiving CAD and autologous implants was 47 and 52 years, respectively. Traumatic brain injury (TBI) (n = 82, 41.0 %) and ischemic stroke (n = 75, 37.5 %) were the most common reasons for DC, while intracerebral hemorrhage (ICH) (n = 38, 19.0 %) and subarachnoid hemorrhage (SAH) (n = 42, 21.0 %) were less common. Fourteen patients (7 %) had died at the time of the 12-month follow-up. CP was performed within 3 months in 37.3 % (n = 69) of the cases. The average number of days between DC and CP was 178.1 ± 220.7 days (Table 1) (Supplemental Digital Content 1).

**Table 1:** Overview of patient (n = 200) characteristics; (DC = decompressive craniectomy, CP = cranioplasty, TBI = traumatic brain injury, ICH = intracranial hemorrhage, ASAH = aneurysmatic subarachnoid hemorrhage, CAD = computer aided design, CRAR = cranioplasty-associated reoperation, FLTO = favorable long-term outcome, ASA = American Society of Anesthesiologists).

Baseline characteristic	N (%)	CRAR			FLTO		
		no (%)	yes (%)	p-value	unfavorable (%)	favorable (%)	p-value
Sex				.010			.206
Female	84 (42)	92 (79)	24 (21)		50 (43)	64 (56)	
Male	116 (58)	58 (69)	26 (31)		48 (57)	36 (43)	

1st implantation				.919			.650
Yes	159 (79.5)	119 (75)	40 (25)		79 (50)	78 (50)	
No	41 (20.5)	31 (76)	10 (24)		19 (46)	22 (54)	

underlying condition				.073			<.001
TBI	82 (41)	60 (73)	22 (27)		24 (30)	57 (70)	
ICH	20 (10)	15 (75)	5 (25)		14 (70)	6 (30)	
Ischemic stroke	72 (36)	60 (83)	12 (17)		48 (68)	23 (32)	
ASAH	26 (13)	15 (58)	11 (42)		12 (46)	14 (54)	

ASA				.001			<.001
1	9 (5)	8 (89)	1 (11)		1 (11)	8 (89)	
2	61 (34)	50 (82)	11 (18)		13 (22)	47 (78)	
3	102 (57)	73 (72)	29 (28)		66 (65)	36 (35)	
4	7 (4)	1 (14)	6 (86)		6 (86)	1 (14)	

medical history & risk factors							
Hypertension	91 (45.5)	64 (70)	27 (30)	.163	57 (64)	32 (36)	<.001
Diabetes	23 (11.5)	17 (74)	6 (26)	.898	19 (83)	4 (17)	<.001
Smoker	33 (16.5)	27 (82)	6 (18)	.322	19 (58)	14 (42)	.309
Alcohol	10 (5)	9 (90)	1 (10)	.261	6 (60)	4 (40)	.495
Wound healing disorder	25 (12.5)	16 (64)	9 (36)	.175	9 (36)	16 (64)	.149
Immunosuppression	7 (3.5)	6 (86)	1 (14)	.505	5 (71)	2 (29)	.237
Coagulation disorder	14 (7)	11 (79)	3 (21)	.749	6 (43)	8 (57)	.606
Other risk factors	41 (20.5)	37 (90)	4 (10)	.011	29 (73)	11 (27)	.001
Coagulation affecting medication	79 (40.9)	65 (82)	14 (18)	.056	51 (66)	26 (34)	<.001
Sinking flap syndrome	24 (12.2)	147 (75)	50 (25)	.145	97 (49)	98 (50)	.086
CP performed within 3 months of DC	69 (37.3)	46 (67)	23 (33)	.028	27 (40)	41 (60)	.037

Type of implant & material				.935			.002
Autologous bone	93 (46.5)	46 (67)	23 (33)		27 (40)	41 (60)	
CAD	107 (53.5)	80 (75)	27 (25)		42 (39)	65 (60)	
Polyetheretherketone	12 (6)	11 (92)	1 (8)		5 (42)	7 (58)	
Polymethylmethacrylate	23 (11.5)	20 (87)	3 (13)		7 (30)	16 (70)	
Ceramic	4 (2)	3 (75)	1 (25)		3 (75)	1 (25)	
Titanium	39 (19.5)	24 (62)	15 (38)		16 (41)	21 (59)	
Hydroxyapatite	7 (3.5)	6 (86)	1 (14)		2 (29)	5 (71)	
Miscellaneous	20 (9)	15 (75)	5 (25)		8 (40)	12 (60)	

### 3.2 Rate of cranioplasty-associated reoperation (CPAR)

**Fig. 1:**
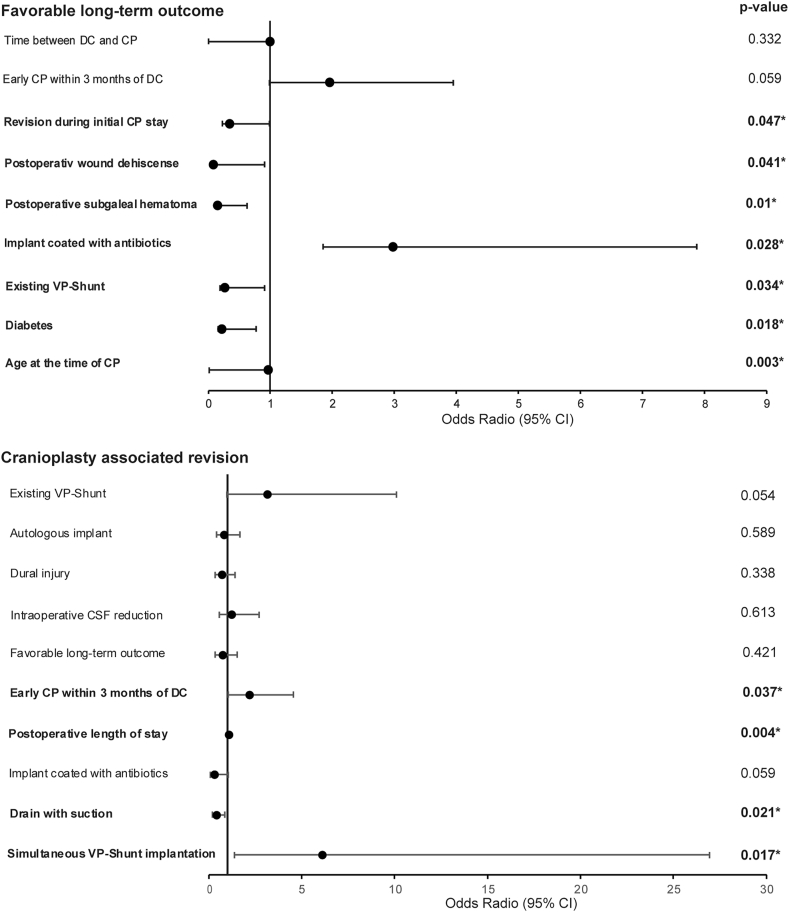
Forest tree plot of the multivariate logistic regression model adjusted for diagnosis, age, and sex: relevant variables assessed in the German Cranial Reconstruction Registry (GCRR) analyzed for their predictive value of the long-term surgical revision rate (CPAR) and favorable long-term outcome (FLTO). (CP – cranioplasty, DC – decompressive craniectomy, VPS – ventriculoperitoneal shunt, CSF – cerebrospinal fluid).

The rate of cranioplasty-associated reoperation (CPAR) in this long-term follow-up was 25 % (n = 50). There was no statistical difference in the surgical revision rate between allogenic and autologous implants. Of the 93 patients who received autologous bone transplants, 23 (25 %) underwent CPAR. Both univariate (p = .935) and multivariate (p = .589) analyses showed no significant correlation between the rate of reoperation and the implant material (p = .441). The most common reasons for CPAR were wound infection (n = 31, 15.6 %), aseptic bone resorption (n = 4, 3.0 %), and ventriculoperitoneal shunt-associated complications (n = 7, 3.5 %) (Table 2). In total, there were 22 (11.0 %) cases of aseptic bone resorption, with a revision rate of 18.2 % (n = 4) within the follow-up period. Neither the perioperative use of single-shot antibiotics (p = .070) nor the antibiotic coating of the implants (p = .057) predicted a higher rate of CPAR. The average duration of CP surgery was 130.7 ± 41.4 min; however, there was no correlation between the duration of surgery and the rate of reoperation (p = .197). Simultaneous VPS implantation (p = .017, 95 % CI, OR 1.387–26.947), early CP within three months of DC (p = .037, 95 % CI, OR 1.049–4.564), and a higher ASA score (p = .031, 95 % CI, OR 1.363–624.342) predicted an increased rate of surgical revision. The use of drains with suction predicted a lower rate of surgical revision (p .037, 95 % CI OR .298–.0964) (Table 3) (Fig. 1).

**Table 2:** Complications requiring surgical revisions in long-term follow-up grouped by mplant type; (CAD – computer aided design).

Implant type	Complication	N (%)
autologous	No revision	69 (75 %)
	wound infection	15 (16 %)
	Resorption	4 (4 %)
	Pneumocephalus	1 (1 %)
	Shunt associated complications/hydrocephalus	3 (3 %)
CAD	No revision	80 (75 %)
	wound infection	18 (17 %)
	Epidural/subdural hematoma	4 (5 %)
	Pneumocephalus	1 (1 %)
	Shunt associated complications/hydrocephalus	4 (4 %)

**Table 3:** Cranioplasty-associated revision: Significant results of the multivariate logistic regression model for independent variables with cranioplasty-associated revision (CPAR) as the dependent variable: All variables were adjusted for age, sex, and diagnosis. ∗represents a statistically significant value of p < .05; (ASA = American society of anesthesiologists, DC = decompressive craniectomy, CP = cranioplasty, TBI = traumatic brain injury, ICH = intracranial hemorrhage, SAH = subarachnoid hemorrhage, VPS = ventriculoperitoneal shunt).

	95 % CI for OR			
	OR	Lower	Upper	p-value
Sex	1.542	.773	3.077	.219
TBI				.087
TBI vs ICH	.710	.222	2.271	.564
TBI vs stroke	.467	.206	1.060	.069
TBI vs SAH	1.651	.637	4.277	.302
Age at the time of CP	1.011	.990	1.033	.302
Simultaneous VPS implantation	6.114	1.387	26.947	**.017**∗
Existing VPS	3.143	.979	10.095	.054
ASA classification				**.022**∗
ASA I vs ASA II	1.270	.138	11.673	.833
ASA I vs ASA III	3.226	.359	29.014	.296
ASA I vs ASA IV	29.173	1.363	624.342	**.031**∗
Drain with suction	.410	.193	.874	**.021**∗
Any complication during hospitalization	6.243	2.798	13.929	< **.001**∗
Early CP within 3 months of DC	2.189	1.049	4.564	**.037**∗
Postoperative length of stay	1.077	1.024	1.133	**.004**∗
Number of drains	.536	.298	.964	**.037**∗

### 3.3 Neurological outcome

**Fig. 2:**
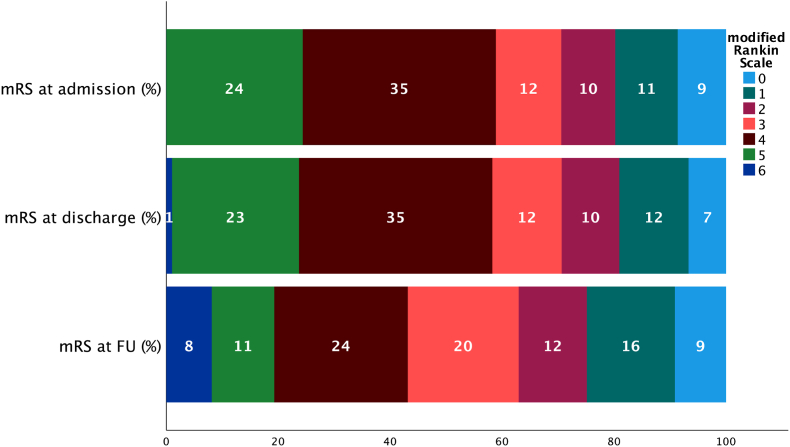
The modified Rankin scale (mRS) at hospital admission for cranioplasty (CP) surgery, discharge and at the follow-up (mean follow-up 938 days), 0 – no symptoms, 1 - no significant disability, 2 – slight disability, 3 – moderate disability, 4 - moderately severe disability, 5- severe disability 6 – dead.

**Fig. 3:**
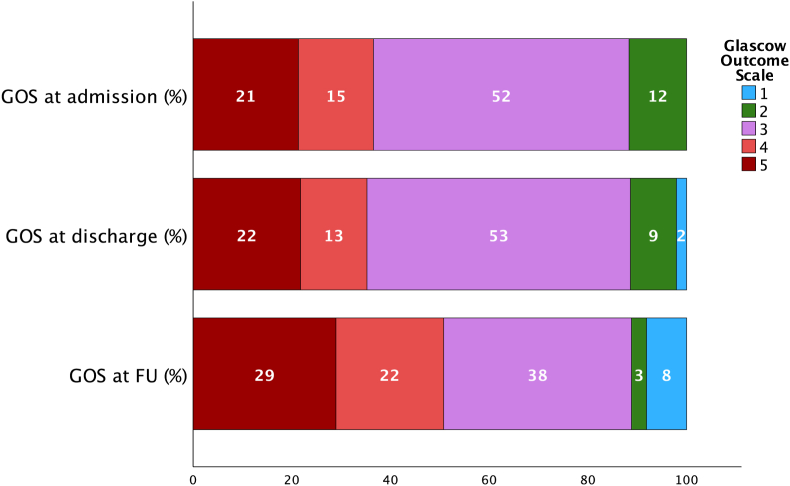
The Glasgow outcome score (GOS) at hospital admission for cranioplasty (CP) surgery, discharge and at the follow-up (mean follow-up 938 days), 1- dead, 2, - neurovegetative state, 3 – severe disability, 4 - moderate disability, 5 – good recovery.

The clinical performance indices indicated that 50 % (n = 100) of patients had favorable long-term outcomes. Of the patients, 37.7 % experienced an improvement in mRS score, and 27.5 % reported an improvement in GOS score (Table 4). In multivariate analysis, TBI was associated with FLTO (p = .001, 95 % CI, OR .127-.532), while an increase in age at the time of CP predicted a poorer functional outcome (p = .003, 95 % CI, OR .950-.990). There was no correlation between FLTO and the reoperation rate (p = .455). An overview of the mRS and GOS scores at admission, discharge, and follow-up is shown in Fig. 2, Fig. 3. Multivariate analysis showed that diabetes mellitus (p = .018, 95 % CI, OR .063-.774) and VPS (p = .034, 95 % CI, OR .077-.905) were variables that predicted unfavorable long-term neurological outcomes (Table 5) (Fig. 1).

**Table 4:** Comparison of the mRS and the GOS at the time of discharge and follow-up.

Score	change	n	%
modified Rankin scale (mRS)	no change	79	39.7 %
	improvement	75	37.7 %
	decline	45	22.6 %

Glascow Outcome scale (GOS)	no change	118	59.0 %
	improvement	55	27.5 %
	decline	27	13.5 %

**Table 5:** Favorable long-term outcome: Significant results of the multivariate logistic regression model for independent variables with favorable long-term outcome (FLTO) as the dependent variable. All variables were adjusted for age, sex, and diagnosis. ∗represents a statistically significant value of p < .05; (ASA = American society of anesthesiologists, CP = cranioplasty, TBI = traumatic brain injury, ICH = intracranial hemorrhage, SAH = subarachnoid hemorrhage).

	OR	95 % CI for OR		
		Lower	Upper	p-value
Sex	1.338	.704	2.542	.374
TBI				**.001**∗
TBI vs ICH	1.663	.643	4.300	.294
TBI vs stroke	.403	.116	1.406	.154
TBI vs SAH	.432	.166	1.120	.084
Age at the time of CP	.969	.950	.990	**.003**∗

Revision during initial CP stay	.347	.122	.985	**.047**∗
diabetes	.221	.063	.774	**.018**∗
Postoperative wound dehiscence	.085	.008	.907	**.041**∗
Autologous implant	1.666	.875	3.171	.120
ASA classification				**.001**∗
ASA I vs ASA II	.483	.048	4.858	.537
ASA I vs ASA III	.115	.012	1.110	.062
ASA I vs ASA IV	.035	.001	.812	**.037**∗
Implant with antibiotics	2.973	1.122	7.876	**.028**∗

## 4 Discussion

The long-term analysis of this prospective cohort study offers a comprehensive assessment of various factors, aimed at guiding the planning, perioperative, and postoperative care of patients who underwent DC. In this study, 50 % of the patients achieved favorable long-term outcomes, whereas 25 % required surgical revision.

An implanted ventriculoperitoneal shunt at the time of CP is a predictor of an increased risk of surgical revision within the initial 30-day period (Sauvigny et al., 2021). However, by applying regression models to patients from the same cohort in the 3-year-follow-up, the existence of a VP shunt failed to predict an increased risk of surgical revision. Owing to underlying pathologies, some patients require VPS placement when CP is indicated. The timing of VPS placement in relation to CP has been under review, with many authors favoring a two-stage procedure (Zhang et al., 2022). A recent Korean study showed a significant risk of VPS and CP complications with simultaneous implantation of both, which was associated with an even higher perioperative risk (Gill et al., 2021). Our study confirmed that simultaneous VPS implantation was predictive of a significant increase in CPAR. Hence, we suggest that these two procedures should be scheduled at two different time points, even if this necessitates additional surgery. The type of valve used in VP shunt surgeries, whether fixed pressure or programmable, was not investigated in this registry.

The role of drains with suction in CP has been a matter of debate in the past, based on insufficient evidence, such as case reports and expert opinions. For instance, there are multiple published cases of patient deaths after CP, in which the authors speculate that the use of drains with suction increases the risk of cerebral edema and death (Broughton et al., 2014; Anuzis et al., 2021). It has been suggested that the negative pressure difference in a large cavity, such as the epidural space, after CP, as well as the resulting brain shift, can cause cerebral edema (Sviri, 2015). The potential benefit of suction drains in CP is likely diminished when dural leaks are detected (Sporns et al., 2016). Others have claimed that this surgical technique minimizes the risk of postoperative epidural hematoma and wound infection (Raju et al., 2023). The results of this prospective study would suggest that the use of drains with suction lowers the overall risk of reoperation without affecting neurological outcomes or mortality. The questionnaire assessed the utilization of drains at the conclusion of surgery. It did not evaluate the placement of the drain (epidural or subgaleal) or the degree of suction applied to the drain. Within this cohort, there was no incidence of brain edema in patients who received suction drains during CP surgery.

The use of CP materials (usually polymethylmethacrylate) coated or imbued with antibiotics seems to predict a more favorable neurological outcome in the regression model of this study without affecting the rate of CPAR in its regression model. We attribute this effect to the small number of patients undergoing CP using antibiotic-coated implants (n = 15). Authors of previously published studies claimed that the antibiotic properties of these implants reduce the rate of postoperative wound infection and therefore lead to overall better neurological recovery (Worm et al., 2016; Lazar et al., 2016). Amendola et al. (2022) published a retrospective cohort study analyzing the antibiotic immersion of CP implants and claimed that this surgical technique led to a significant decrease in postoperative wound infection.

Corallo et al. (2021) showed a significant improvement in neuropsychological and cognitive abilities after CP in patients with TBI. While this study showed an overall improvement of the neurological outcome after CP, there was also a decline in mRS in 25 % and GOS in 12 % of the cases, respectively. A prospective Spanish study demonstrated an association between the timepoint after DC and the measurement of daily autonomy and age (Paredes et al., 2015). The authors observed an objective improvement in the Barthel index, an index used to assess autonomy in activities of daily living, by 40 % within days after CP. The findings of this study show an improvement in GOS and mRS scores of 7.5 % and 37.7 % after CP, respectively. Peredes et al. (Paredes et al., 2015) described a trend in which patients with TBI showed a higher degree of neurological improvement after CP, a finding that was also confirmed by our multivariate logistic regression model. This study did not show an association between implant type and long-term neurological outcomes after CP, independent of the patients’ age and diagnosis. This is despite the fact that neurosurgeons commonly recommend autologous implants for older patients, despite the risk of resorption and the associated risk of reoperation due to reduced life expectancy (Malcolm et al., 2018a).

The process of aseptic bone resorption after CP administration is not fully understood (Göttsche et al., 2021). A recent systematic review of autologous bone resorption showed that bone flap fragmentation, TBI, and younger age increase the risk of bone resorption (Signorelli et al., 2022). Neurosurgeons tend to recommend autologous cranioplasty for patients who accept the risk of bone resorption for a lower infection rate. Thus, non-autologous CP is often recommended for younger patients with greater life expectancy. The authors of a 2017 meta-analysis described a trend of lower explantation rates when comparing CAD implants and autologous bone grafts. They determined a CAD implant failure rate of 8–31 % (Punchak et al., 2017). However, there was no significant increase in the surgical revision rate and no effect on the long-term neurological outcome when comparing cases of autologous and non-autologous implants in this study.

Yang et al. (2018) published the results of a retrospective multicenter study including patients who underwent CP after DC for TBI. The authors argued in favor of early CP within 34 days after DC and showed a correlation between early CP and an improved 6-month GOS. In TBI, early CP is associated with increased cerebral perfusion (Rynkowski et al., 2021). A 2018 meta-analysis indicated that early CP was associated with greater neurological improvement than that with delayed CP (Malcolm et al., 2018b). Vreeburg et al. suggested that early CP, even within the initial hospital stay, in which DC was performed, does not have an increase in rate of complication (Vreeburg et al., 2024). This study, however, suggests that patients who underwent CP within 90 days had a higher revision rate than patients who underwent late CP without a significant impact on neurological outcomes. The healing of the surgical wound after DC, stabilization of CSF fluid dynamics, and recovery from the underlying disease that led to DC may play a role in favoring delayed CP. The economic feasibility of countries such as Germany and Austria, which can finance lengthy rehabilitation processes for patients, may also play a part in this debate. Lastly, in this study, there was no correlation between the syndrome of the trephine and the timing of CP.

### 4.1 Limitations

To our knowledge, this is the only published prospective multicenter study with a follow-up of multiple years after CP. The strength of this study lies in its prospective and multicenter-based design. Unfortunately, more than half of all patient cases were lost to long-term follow-up and therefore had to be excluded. The low mortality rate is likely also linked to loss to follow-up. The heterogeneity of surgical practice, not only among neurosurgical departments but also among surgeons, can be a confounding variable in our study. Although this study is multinational, it only represents common practices in German-speaking countries, Austria and Germany.

## 5 Conclusion

This prospective multicenter study suggests that the use of drains with suction is associated with a lower long-term rate of surgical revision after CP, whereas simultaneous VPS implantation increases the risk of surgical revision. Antibiotic coating of implants predicted a FLTO.

## Statement

During the preparation of this work, the authors used Microsoft Copilot and Paperpal in order to increase fluency, grammar and spelling of the text. After using this tool, the authors reviewed and edited the content as needed and take full responsibility for the content of the publication.

## Declaration of conflicting interests

The German cranial reconstruction registry was funded by 3di GmbH, Skulle implants, OssDsign, 10.13039/100008127DePuy Synthes Spine and the ZNS – 10.13039/501100007731Hannelore Kohl Stiftung.

The authors have no other conflicts to disclose.

## Appendix A Supplementary data

Supplemental **Digital Content 1** Case report forms (CRF) for Cranioplasty and postoperative monitoring forms

Multimedia component 1 Multimedia component 1

**Supplement Digital Content 2:** Univariate analysis of all variables within the German Cranial Reconstruction Registry (GCRR) questionnaire, multivariate logistic regression for CPAR and FLTO of variables with statistical relevance in univariate analysis.

## Footnotes

1 **Abbreviations** DC = decompressive craniectomy, CP = cranioplasty, TBI = traumatic brain injury, ICH = intracranial hemorrhage, ASAH = aneurysmatic subarachnoid hemorrhage, CAD = computer aided design, CPAR = cranioplasty-associated reoperation, FLTO = favorable long-term outcome, CPAR = cranioplasty-associated revision, ASA = American society of anesthesiologists, VPS = ventriculoperitoneal shunt, mRS = modified Rankin scale, GOS = Glasgow outcome score, CRF = case report forms.
