# Supplementary material for: German cranial reconstruction registry – a prospective multicenter cohort study: 883-day follow-up on the outcome and complications
Source: Brain Spine. 2025 Jul 2;5:104308. doi: 10.1016/j.bas.2025.104308 (PMC12272473; doi:10.1016/j.bas.2025.104308)
Supplement: Multimedia component 1 [file mmc1.pdf]

Supplementary Data: Case Report Forms (CRFs) cranioplasty and postoperative monitoring

CRF Part 2

Number of cranioplasty |__|__|

Patienten ID |__|__|__| - |__|__|__|__|
Center — Patient No.

Patient data

Sex ☐ male ☐ female Year of birth |__|__|__|__|

Date of admission |__|__| |__|__| |__|__|__|__| (dd/mm/yyyy)

Current procedure is the first cranioplasty ☐ yes ☐ no

if no: Number of previous attempts |__|

Reason for repeated surgery
☐ Bone flap resorption
☐ Loosening
☐ Infection
☐ Miscellaneous ____________________

Previous surgeries

Patient already registered in GCRR ☐ yes ☐ no

if no: ☐ First surgery (e.g. bony tumor)
Diagnose ____________________
☐ Prior craniectomy
pleas fill out following sections

OP-Diagnosis *(if not already documeted)*

TBI	☐ yes	☐ no	if yes	☐ open	☐ closed
ICH	☐ yes	☐ no	if yes	☐ traumatic	☐ Non-traumatic
Stroke	☐ yes	☐ no	if yes	☐ MCA stroke	☐ ICA stroke
SAH	☐ yes	☐ no	if yes	☐ traumatic	☐ aneurysmatic
miscellaneous	☐ yes	☐ no	if yes, specify	____________________	

Operationsdetails *(if not already documented)*

Date |__|__| |__|__| |__|__|__|__| (tt/mm/jjjj) Skin incision |__|__|:|__|__| (hh/min) Skin sutrue |__|__|:|__|__| (hh/min)

Localisation ☐ right ☐ left ☐ bifrontal ☐ miscellaneous

Type of skin incision

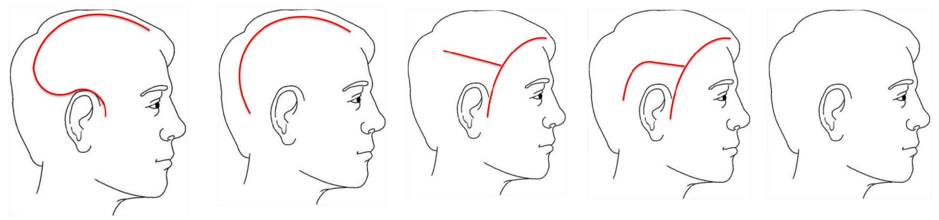

☐ Skin incision-arc shaped ☐ c-shaped ☐ t-shaped ☐ lambda shaped incision ☐ miscellaneous (einzeichnen)

Number of bone fragments |__|

Number of cranioplasty |__|__|

Patienten ID |__|__|__| - |__|__|__|__|
Center Patient No.

Status at time of hospitalisation

Known risk factors ☐ yes ☐ no

if yes:
☐ hypertension ☐ Diabetes
☐ nicotine ☐ alcohol
☐ wound infection ☐ previous wound heeling disorder
☐ Immune suppression ☐ multidrug resistant bacteria
☐ bleeding disorder ____________________
☐ misc. ____________________

Anticoagulants or anti platelet drugs ☐ yes ☐ no

if yes, specify ____________________ Paused ☐ yes ☐ no

Neurological Scores

mRS |__|

GOS |__|

Sinking Skin Flap Syndrome ☐ yes ☐ no

Craniotomy defect ☐ into skull level ☐ elevated ☐ collapsed

VP/VA-Shunt ☐ yes ☐ no, if yes: ☐ Ipsilateral ☐ Contralateral

Anti-epileptic drug ☐ yes ☐ no

if yes, specify ____________________

Implantat

Largest horizontal diameter |__|__|__| mm Largest vertical diameter |__|__|__| mm

Type of implantat

Manufacturing ☐ Autologous ☐ CAD/CAM manufractured ☐ Manually manufractured

Material ☐ PEEK ☐ PMMA ☐ Ceramic
☐ Titanium ☐ Hydroxylapatit
☐ Miscellaneous ____________________

if CAD: the first surgeon was entrusted with the planning of the CAD ☐ yes ☐ no

Antibiotic-coated implant ☐ yes ☐ no

Reason if autologous bone was not used

☐ Discarded ☐ Necrosis ☐ Miscellaneous ____________________

Surgery

Date |__|__| |__|__| |__|__|__|__| (tt/mm/jjjj) Skin incision |__|__|:|__|__| (hh/min) Skin suture |__|__|:|__|__| (hh/min)

ASA classification |__|

Number of surgeons |__|

Expertise of first surgeon

Number of decompressive craniectomies ☐ <10 ☐ 11-40 ☐ >40

Years of neurosurgical practice ☐ <1 ☐ 1-2 ☐ 3-4 ☐ 5-6 ☐ 7-8 ☐ >8

CRF Part 2

Number of cranioplasty |__|__|

Patienten ID |__|__|__| - |__|__|__|__|
Center Patient No.

Surgery

Opening of frontal sinus ☐ yes ☐ no

Withdrawal of CSF ☐ yes ☐ no

if yes: ☐ Cannulation of the ventricle ☐ Puncture of CSF-reservoir ☐ Lumbar drainage

Simultaneous Shunt implantation ☐ yes ☐ no

Skin expander ☐ yes ☐ no

Intraoperative adjustment ☐ yes ☐ no

Fixation

Clamps	Number |__|__|	☐ absorbable	☐ non-absorbable
Plates	Number |__|__|	☐ absorbable	☐ non-absorbable
Sitches	Number |__|__|	☐ absorbable	☐ non-absorbable
Misc.	______________________			

Dural tenting sutures ☐ yes ☐ no if yes, number |__|__|

Dural injury ☐ yes ☐ no

Anatomical reposition of the temporalis muscle ☐ yes ☐ no

Wound drainage

Number |__| Size of drains |__|__| Charriére (CH)

Drain with suction ☐ yes ☐ no

Skin closure ☐ Skin-clamps ☐ Suture

if suture: Material ☐ absorbable ☐ non-absorbable

Type of sutures ☐ Continuous suture ☐ Intermittent suture ☐ Horizontal/vertical suture

Impeded skin closure ☐ yes ☐ no

Wound crosses edge of craniotomy ☐ yes ☐ no

Perioperative prophylactic antibiotics

Single-Shot ☐ yes ☐ no if yes, type ______________________

Postoperative continued antibiotic medication ☐ yes ☐ no if yes, type ______________________

Duration |__|__| days

Estimated blood loss |__|__|__|__| ml

Intraoperative complications ☐ yes ☐ no

if yes, specify: ______________________

CRF Part 3

Number of postoperative follow-up |__|__|

Patient ID |__|__|__| - |__|__|__|__|
Center Patient No.

Patient data

Sex ☐ male ☐ female Year of birth |__|__|__|__|

Postoperative course

Postoperative care on ICU ☐ yes ☐ no if yes, for |__|__| hours

Postoperative CT-scan ☐ yes ☐ no if yes, for |__|__| hours

Removal of drains |__|__| postoperative days

Removal of suture materials |__|__| postoperative days

Postoperative complications until discharge ☐ yes ☐ no

if yes:	yes	no	Treatment: no	Conservative	Explantation of cranioplasty	Miscellaneous surgery
New ischemic areal	☐	☐	☐	☐	☐	☐
CSF fistula	☐	☐	☐	☐	☐	☐
Seizures	☐	☐	☐	☐	☐	☐
New hydrocephalus	☐	☐	☐	☐	☐	☐
Subdural hematoma	☐	☐	☐	☐	☐	☐
Epidural hematoma	☐	☐	☐	☐	☐	☐
Subgaleal hematoma	☐	☐	☐	☐	☐	☐
Meningitis / Ventriculitis	☐	☐	☐	☐	☐	☐
CSF leak	☐	☐	☐	☐	☐	☐
Wound infection	☐	☐	☐	☐	☐	☐
Wound dehiscence	☐	☐	☐	☐	☐	☐
Mis. complications	☐	☐	☐	☐	☐	☐

____

if explantation of cranioplasty, Date of explantation |__|__| |__|__| |__|__|__|__|
(dd/mm/yyy)

Discharge

Patient died ☐ yes ☐ no if yes, Date of Death |__|__| |__|__| |__|__|__|__|
(dd/mm/yyyy)

if no, Date of discharge |__|__| |__|__| |__|__|__|__|
(dd/mm/yyyy)

Discharged to ☐ Rehabilitation clinic, Phase ☐ B ☐ C ☐ External hospital
☐ Rest home ☐ Home

Neurological scores at discharge

mRS |__|

GOS |__|
