# Supplementary material for: German cranial reconstruction registry – a prospective multicenter cohort study: 883-day follow-up on the outcome and complications
Source: Brain Spine. 2025 Jul 2;5:104308. doi: 10.1016/j.bas.2025.104308 (PMC12272473; doi:10.1016/j.bas.2025.104308)
Supplement: Multimedia component 2 [file mmc2.docx]

| **Variable** | **n** | **Yes** | **%** | **No** | **%** | **Surigcal revision** | **Long-term favorable outcome** |
| --- | --- | --- | --- | --- | --- | --- | --- |
| Sex (male, female) | 200 | 116 | 58,0 | 84 | 42,0 | 0.098 | **0.044** |
| Traumatic brain injury | 200 | 82 | 41,0 | 118 | 59,0 | 0.618 | <.001* |
| Intracranial hemorrhage | 200 | 38 | 19,0 | 162 | 81,0 | 0.298 | 0.470 |
| Stroke | 200 | 75 | 37,5 | 125 | 62,5 | **0.023** | **<.001*** |
| Subarachnoidal hemorrhage | 200 | 42 | 21,0 | 158 | 79,0 | 0.071 | 0.082 |
| Any risk factor | 200 | 149 | 74,5 | 51 | 25,5 | 0.303 | **.002** |
| Hypertension | 200 | 91 | 45,5 | 109 | 54,5 | 0.163 | **<.001*** |
| Diabetes | 200 | 23 | 11,5 | 177 | 88,5 | 0.898 | **<.001*** |
| Smoker | 200 | 33 | 16,5 | 167 | 83,5 | 0.322 | 0.340 |
| Alcohol | 200 | 10 | 5,0 | 190 | 95,0 | 0.261 | 0.516 |
| History of wound infection | 200 | 9 | 4,5 | 191 | 95,5 | 0.555 | 0.731 |
| Immunosuppression | 200 | 7 | 3,5 | 193 | 96,5 | 0.505 | 0.248 |
| Coagulation disorder | 200 | 14 | 7,0 | 186 | 93,0 | 0.749 | 0.579 |
| Coagulation medication | 193 | 79 | 40,9 | 114 | 59,1 | 0.056 | **<.001*** |
| Coagulation medication paused at the time of surgery | 200 | 71 | 35,5 | 129 | 64,5 | 0.201 | **0.002** |
| Sinking flap syndrome | 197 | 24 | 12,2 | 173 | 87,8 | 0.145 | 0.086 |
| Protruding soft tissue | 198 | 42 | 21,2 | 156 | 78,8 | 0.875 | 0.263 |
| Existing ventriculoperitoneal shunt | 197 | 16 | 8,1 | 181 | 91,9 | 0.002 | 0.118 |
| Anti-epileptic drugs | 190 | 63 | 33,2 | 127 | 66,8 | 0.043 | 0.916 |
| Implant type (autologous, CAD) | 200 | 93 | 46,5 | 107 | 53,5 | 0.935 | **0.003** |
| Intraoperative CSF reduction | 199 | 58 | 29,1 | 141 | 70,9 | 0.643 | **0.013** |
| Implant coated with antibiotics | 185 | 27 | 14,6 | 158 | 85,4 | 0.057 | **0.028** |
| Surgeon planned CAD | 104 | 37 | 35,6 | 67 | 64,4 | **0.031** | 0.886 |
| Intraoperative opening of frontal sinus | 200 | 2 | 1,0 | 198 | 99,0 | 0.412 | 0.155 |
| Use of skin expander | 195 | 3 | 1,5 | 192 | 98,5 | 0.305 | 0.079 |
| Simultaneous VP-Shunt implantation | 200 | 9 | 4,5 | 191 | 95,5 | **0.003** | 0.088 |
| Intraoperative change of implants | 199 | 31 | 15,6 | 168 | 84,4 | **0.031** | 0.821 |
| Dura-holding stiches | 197 | 167 | 84,8 | 30 | 15,2 | 0.528 | 0.714 |
| Dural injury | 193 | 82 | 42,5 | 111 | 57,5 | 0.346 | **0.047** |
| Anatomical repositioning of temporal muscle | 188 | 143 | 76,1 | 45 | 23,9 | 0.235 | 0.235 |
| Drain with suction | 182 | 127 | 69,8 | 55 | 30,2 | **0.016** | 0.093 |
| Single shot antibiotic | 198 | 193 | 97,5 | 5 | 2,5 | 0.07 | 0.651 |
| Skin closure with staples or stiches | 197 | 23 | 11,7 | 174 | 88,3 | 0.458 | 0.384 |
| Post-op antibiotic prophylaxis | 165 | 45 | 27,3 | 120 | 72,7 | 0.492 | 0.600 |
| Post-op ICU | 195 | 94 | 48,2 | 101 | 51,8 | 0.432 | 0.993 |
| Post-op cCT | 194 | 158 | 81,4 | 36 | 18,6 | 0.082 | 0.918 |
| Wound dehiscence | 200 | 5 | 2,5 | 195 | 97,5 | **<.001*** | 0.174 |
| Wound infection | 200 | 19 | 9,5 | 181 | 90,5 | **<.001*** | 0.809 |
| Intraoperative complications | 199 | 8 | 4,0 | 191 | 96,0 | 0.401 | 0.988 |
| Any complication during hospital stays | 191 | 53 | 27,7 | 138 | 72,3 | **<.001*** | 0.091 |
| Ischemia | 199 | 3 | 1,5 | 196 | 98,5 | 0.737 | 0.081 |
| CSP fistula | 200 | 4 | 2,0 | 196 | 98,0 | 1 | 1 |
| Postoperative seizure | 200 | 6 | 3,0 | 194 | 97,0 | 0.632 | 0.097 |
| New hydrocephalus | 200 | 7 | 3,5 | 193 | 96,5 | **<.001*** | 0.700 |
| Subdural hematoma | 200 | 11 | 5,5 | 189 | 94,5 | 0.371 | 0.352 |
| Epidural hematoma | 200 | 17 | 8,5 | 183 | 91,5 | **<.001*** | 0.447 |
| Subgaleal hematoma | 200 | 11 | 5,5 | 189 | 94,5 | 0.107 | 0.352 |
| Meningitis/ventriculitis | 200 | 2 | 1,0 | 198 | 99,0 | **0.014** | 0.155 |
| CSF Leak | 200 | 4 | 2,0 | 196 | 98,0 | 0.243 | 0.312 |
| Misc complication | 200 | 17 | 8,5 | 183 | 91,5 | 0.305 | 0.076 |
| Surgical revision | 200 | 150 | 75,0 | 50 | 25,0 |  | 0.41 |
| GOS improvement | 200 | 55 | 27,5 | 145 | 72,5 | 0.492 | **<.001*** |
| mRS improvement | 199 | 75 | 37,7 | 124 | 62,3 | 0.445 | **<.001*** |
| **Table 1:** univariate analysis for variables long-term favorable outcome and reoperation. Statistical analysis was done using the chi-squared test or Fisher’s exact test when appropriate. A p-value of <0.05 was deemed statistically significant and highlighted. | | | | | | | |

|  | | Mann-Whitney U | | Wilcoxon W | | Z | | Asymp. Sig. (2-tailed) |
| --- | --- | --- | --- | --- | --- | --- | --- | --- |
| **Days between surgery and FU** | | **2.913.000** | | **13.644.000** | | **-2.129** | | **.033** |
| **Days between DC and CP** | | **2.427.000** | | **3.462.000** | | **-2.314** | | **.021** |
| Blood loss per duration of surgery | | 1.963.000 | | 2.593.000 | | -.515 | | .606 |
| Defect size - calculated | | 3.074.500 | | 14.100.500 | | -1.786 | | .074 |
| **Length of stay** | | **1.318.500** | | **10.229.500** | | **-5.021** | | **<.001** |
| **Postoperative stay at hospital** | | **1.856.000** | | **11.447.000** | | **-4.082** | | **<.001** |
| Length of ICU stay | | 634.000 | | 887.000 | | -.612 | | .540 |
| Post-op CT scan | | 2.113.500 | | 3.059.500 | | -1.259 | | .208 |
| Blood loss in ml | | 3.160.000 | | 12.476.000 | | -.116 | | .908 |
| Number of staples | | 1.643.000 | | 2.238.000 | | -1.825 | | .068 |
| Number of tiles | | 2.716.500 | | 3.662.500 | | -.206 | | .837 |
| Number of stiches | | 1.603.000 | | 2.164.000 | | -.444 | | .657 |
| **Number of drains** | | **2.874.000** | | **4.149.000** | | **-2.614** | | **.009** |
| Size of drain in CH | | 2.781.000 | | 12.097.000 | | -.028 | | .977 |
| Number of operating surgeons | | 3.515.000 | | 14.840.000 | | -.819 | | .413 |
| Number of CP of first surgeon | | 3.596.500 | | 14.921.500 | | -.469 | | .639 |
| Neurosurgical years of first surgeon | | 3.540.500 | | 14.865.500 | | -.604 | | .546 |
| Duration of CP in minutes | | 3.492.000 | | 4.767.000 | | -.728 | | .467 |
| Age at CP | | 3.494.000 | | 14.819.000 | | -.723 | | .470 |
| **Table 2**: univariate analysis for independent numerical factors and reoperation. Statistical analysis was done using the student t-tests or the Wilcoxon-Mann-Whitney-Test when appropriate. A p-value of <0.05 was deemed statistically significant and highlighted. | | | | | | | | |
|  |  | |  | |  | |  | |
|  | | | | | | | | |

|  | Mann-Whitney U | Wilcoxon W | Z | Asymp. Sig. (2-tailed) |
| --- | --- | --- | --- | --- |
| **Days between surgery and FU** | 4.263.500 | 8.823.500 | -1.123 | .261 |
| **Days between DC and CP** | **3.405.000** | **7.591.000** | **-2.180** | **.029** |
| Blood loss per duration of surgery | 2.637.500 | 5.193.500 | -.879 | .379 |
| Defect size - calculated | 4.519.500 | 9.370.500 | -.711 | .477 |
| **Length of stay** | 3.455.500 | 7110.500b | -.869 | .385 |
| **Postoperative stay at hospital** | 4.051.500 | 8.329.500 | -.251 | .802 |
| Length of ICU stay | **591.000** | **1.537.000** | **-2.836** | **.005** |
| Post-op CT scan | 2.726.000 | 5.966.000 | -.985 | .324 |
| Blood loss in ml | **3.273.500** | **7.368.500** | **-2.351** | **.019** |
| Number of staples | **2.211.500** | **4.912.500** | **-2.253** | **.024** |
| Number of tiles | **2.763.000** | **6.679.000** | **-2.802** | **.005** |
| Number of stiches | 2.087.000 | 3.978.000 | -.611 | .541 |
| **Number of drains** | 4.308.000 | 9.061.000 | -1.283 | .199 |
| Size of drain in CH | **3.234.500** | **7.062.500** | **-2.062** | **.039** |
| Number of operating surgeons | 4.657.000 | 9.607.000 | -.748 | .455 |
| Number of CP of first surgeon | 4.625.000 | 9.575.000 | -.740 | .459 |
| Neurosurgical years of first surgeon | 4.887.500 | 9.837.500 | -.033 | .974 |
| Duration of CP in minutes | 4.708.500 | 9.658.500 | -.476 | .634 |
| Age at CP | **3.314.000** | **8.264.000** | **-3.937** | **<.001** |
| **Table 3**: univariate analysis for independent numerical factors and LTFO. Statistical analysis was done using the student t-tests or the Wilcoxon-Mann-Whitney-Test when appropriate. A p-value of <0.05 was deemed statistically significant and highlighted. | | | | |

|  | | Sig. | OR | 95% CI for OR | |
| --- | --- | --- | --- | --- | --- |
|  |  |  |  | Lower | Upper |
|  | Sex | .374 | .747 | .393 | 1.420 |
|  | **TBI** | **.001** |  |  |  |
|  | TBI vs ICH | **.012** | .242 | .080 | .733 |
|  | TBI vs stroke | **<.001** | .260 | .127 | .532 |
|  | TBI vs SAH | .294 | .601 | .233 | 1.555 |
|  | **Age at the time of CP** | **.003** | **.969** | **.950** | **.990** |
|  | Any risk factor | .225 | .611 | .276 | 1.353 |
|  | Hypertension | .161 | .610 | .305 | 1.218 |
|  | **diabetes** | **.018** | **.221** | **.063** | **.774** |
|  | Smoker | .216 | .593 | .260 | 1.355 |
|  | Alcohol | .766 | .809 | .200 | 3.278 |
|  | History of wound infection | .921 | .926 | .202 | 4.249 |
|  | Previous wound healing disorder | .255 | 1.748 | .668 | 4.572 |
|  | Immunosuppression | .302 | .404 | .072 | 2.257 |
|  | **History of multi-resistant bacteria** | **.001** | **.075** | **.015** | **.361** |
|  | Coagulation disorder | .410 | 1.691 | .485 | 5.898 |
|  | **Misc. Risk factors** | **.023** | **.381** | **.166** | **.875** |
|  | Coagulation medication | .136 | .579 | .282 | 1.188 |
|  | Coagulates paused | .154 | .595 | .291 | 1.215 |
|  | **Favorable mRS at admission** | **<.001** | **8.735** | **3.941** | **19.359** |
|  | **Favorable GOS at admission** | **<.001** | **16.613** | **6.443** | **42.836** |
|  | Sinking flap syndrome | .094 | .427 | .158 | 1.155 |
|  | bulging flap | .314 | .671 | .309 | 1.458 |
|  | **Existing VP-shunt** | **.034** | **.265** | **.077** | **.905** |
|  | Antiepileptic drugs | .055 | .476 | .223 | 1.015 |
|  | Defect size | .204 | 1.000 | 1.000 | 1.000 |
|  | Autologous implant | .120 | 1.666 | .875 | 3.171 |
|  | Implant material (PEEK baseline) | .266 |  |  |  |
|  | PEEK vs PMMA | .127 | 4.078 | .670 | 24.812 |
|  | PEEK vs ceramic | .253 | .186 | .010 | 3.314 |
|  | PEEK vs titan | .437 | 1.874 | .384 | 9.136 |
|  | PEEK vs hydroxy | .207 | 4.390 | .441 | 43.702 |
|  | PEEK vs misc | .686 | 1.428 | .254 | 8.025 |
|  | Surgeon planned implant | .880 | .930 | .364 | 2.379 |
|  | **Implant with antibiotics** | **.028** | **2.973** | **1.122** | **7.876** |
|  | **ASA classification** | **.001** |  |  |  |
|  | ASA I vs ASA II | .537 | .483 | .048 | 4.858 |
|  | ASA I vs ASA III | .062 | .115 | .012 | 1.110 |
|  | **ASA I vs ASA IV** | **.037** | **.035** | **.001** | **.812** |
|  | Number of surgeons | .679 | 1.134 | .625 | 2.056 |
|  | Intraoperative CSF reduction | .226 | 1.563 | .759 | 3.220 |
|  | Number of CP performed by first surgeon | .265 | .777 | .498 | 1.211 |
|  | Number of years of the first neurosurgeon | .308 | .896 | .726 | 1.106 |
|  | Duration of CP (in min) | .226 | .995 | .987 | 1.003 |
|  | Opening frontal sinus | .999 | .000 | .000 |  |
|  | Simultaneous shunt implantation | .088 | .255 | .053 | 1.228 |
|  | Intraoperative change of implants | .936 | .965 | .402 | 2.316 |
|  | Dura-holding stiches | .376 | 1.470 | .626 | 3.449 |
|  | Dural injury | .125 | .610 | .324 | 1.148 |
|  | Anatomical repositioning of temporal muscle | .221 | .630 | .300 | 1.320 |
|  | Number of staples | .345 | .940 | .827 | 1.069 |
|  | **Number of tiles** | **.035** | **.845** | **.722** | **.988** |
|  | Number of drains | .476 | .823 | .482 | 1.405 |
|  | Drain with suction | .175 | .605 | .293 | 1.249 |
|  | **Challenging skin closure** | **.024** | **.319** | **.118** | **.860** |
|  | **Scar crosses craniotomy** | **<.001** | **.253** | **.120** | **.533** |
|  | Intraoperative single shot antibiotics | .872 | 1.175 | .165 | 8.352 |
|  | Postoperative prophylactic antibiotic therapy | .993 | .997 | .465 | 2.134 |
|  | Blood loss | .267 | .999 | .998 | 1.001 |
|  | Intraoperative complications | .883 | 1.128 | .227 | 5.606 |
|  | Postoperative ICU stay | .983 | 1.007 | .534 | 1.897 |
|  | Postoperative cranial CT | .307 | .655 | .291 | 1.475 |
|  | Any complication during hospitalization | .008 | .349 | .161 | .756 |
|  | Postoperative ischemia | .999 | .000 | .000 |  |
|  | Postoperative CSF fistula | .745 | 1.415 | .174 | 11.480 |
|  | Postoperative seizure | .226 | .250 | .026 | 2.358 |
|  | New postoperative hydrocephalus | .846 | .849 | .162 | 4.438 |
|  | Postoperative subdural hematoma | .457 | .598 | .154 | 2.318 |
|  | Postoperative epidural hematoma | .135 | .410 | .128 | 1.319 |
|  | **Postoperative subgaleal hematoma** | **.010** | **.146** | **.034** | **.628** |
|  | Postoperative meningitis | .999 | .000 | .000 |  |
|  | Postoperative CSF leak | .271 | .240 | .019 | 3.047 |
|  | Postoperative wound infection | .607 | .762 | .271 | 2.147 |
|  | **Postoperative wound dehiscence** | **.041** | **.085** | **.008** | **.907** |
|  | Miscellaneous postoperative complication | .100 | .377 | .118 | 1.205 |
|  | Postoperative length of stay | .662 | 1.003 | .990 | 1.016 |
|  | CP performed before 1pm | .051 | 1.874 | .996 | 3.527 |
|  | Time between DC and CP | .332 | .999 | .998 | 1.001 |
|  | Early CP within 3 months of DC | .059 | 1.960 | .974 | 3.945 |
|  | **Already revision during initial stay** | **.047** | **.347** | **.122** | **.985** |
|  | Complications without reoperation | .718 | 1.148 | .543 | 2.426 |
|  | Resorption of autologous implant | .598 | .776 | .302 | 1.993 |
|  | Reoperation | .455 | .759 | .368 | 1.565 |
| **Table 4**: multivariate binary logistic regression for long-term favorable outcome with Odds ratio and 95% confidence intervals, CP – cranioplasty, DC – decompressive craniectomy, TBI – traumatic brain injury, ICH – intracranial hemorrhage, SAH – subarachnoid hemorrhage, OR – odds ratio, CI – confidence interval* represents a statistically significant value of p < 0.05 | | | | | |

|  | | Sig. | OR | 95% CI for OR | |
| --- | --- | --- | --- | --- | --- |
|  |  |  |  | Lower | Upper |
|  | Sex | .219 | 1.542 | .773 | 3.077 |
|  | TBI | .087 |  |  |  |
|  | TBI vs ICH | .564 | .710 | .222 | 2.271 |
|  | TBI vs stroke | .069 | .467 | .206 | 1.060 |
|  | TBI vs SAH | .302 | 1.651 | .637 | 4.277 |
|  | Age at the time of CP | .302 | 1.011 | .990 | 1.033 |
|  | Any risk factor | .428 | 1.425 | .593 | 3.428 |
|  | Hypertension | .219 | 1.623 | .750 | 3.514 |
|  | diabetes | .770 | .844 | .272 | 2.623 |
|  | Smoker | .366 | .635 | .238 | 1.698 |
|  | Alcohol | .418 | .416 | .050 | 3.480 |
|  | History of wound infection | .349 | 2.006 | .467 | 8.617 |
|  | Previous wound healing disorder | .112 | 2.152 | .836 | 5.539 |
|  | Immunosuppression | .497 | .467 | .052 | 4.199 |
|  | History of multi-resistant bacteria | .365 | 1.622 | .569 | 4.624 |
|  | Coagulation disorder | .906 | .920 | .232 | 3.655 |
|  | Misc. Risk factors | .028 | .288 | .095 | .874 |
|  | Coagulation medication | .906 | .920 | .232 | 3.655 |
|  | Coagulates paused | .501 | .761 | .343 | 1.687 |
|  | Favorable mRS at admission | .009 | .309 | .128 | .747 |
|  | Favorable GOS at admission | .010 | .288 | .111 | .743 |
|  | Sinking flap syndrome | .354 | 1.569 | .605 | 4.066 |
|  | bulging flap | .556 | 1.276 | .566 | 2.879 |
|  | Existing VP-shunt | .054 | 3.143 | .979 | 10.095 |
|  | Antiepileptic drugs | .137 | 1.736 | .839 | 3.594 |
|  | Defect size | .068 | 1.000 | 1.000 | 1.000 |
|  | Autologous implant | .589 | .824 | .408 | 1.662 |
|  | Implant material (PEEK baseline) | .172 |  |  |  |
|  | PEEK vs PMMA | .778 | 1.431 | .118 | 17.287 |
|  | PEEK vs ceramic | .314 | 5.173 | .211 | 126.713 |
|  | PEEK vs titan | .086 | 7.042 | .758 | 65.398 |
|  | PEEK vs hydroxy | .900 | 1.217 | .056 | 26.350 |
|  | PEEK vs misc | .248 | 4.086 | .374 | 44.613 |
|  | **Surgeon planned implant (n=104)** | **.042** | **.316** | **.104** | **.961** |
|  | Implant with antibiotics | .059 | .293 | .082 | 1.046 |
|  | **ASA classification** | **.022** |  |  |  |
|  | ASA I vs ASA II | .833 | 1.270 | .138 | 11.673 |
|  | ASA I vs ASA III | .296 | 3.226 | .359 | 29.014 |
|  | **ASA I vs ASA IV** | **.031** | **29.173** | **1.363** | **624.342** |
|  | Number of surgeons | .398 | 1.314 | .698 | 2.474 |
|  | Intraoperative CSF reduction | .613 | 1.225 | .558 | 2.691 |
|  | Number of CP performed by first surgeon | .913 | 1.027 | .638 | 1.653 |
|  | Number of years of the first neurosurgeon | .748 | 1.037 | .829 | 1.299 |
|  | Duration of CP (in min) | .449 | .997 | .989 | 1.005 |
|  | Opening frontal sinus | .497 | 2.726 | .151 | 49.226 |
|  | **Simultaneous shunt implantation** | **.017** | **6.114** | **1.387** | **26.947** |
|  | Intraoperative change of implants | .055 | .292 | .083 | 1.027 |
|  | Dura-holding stiches | .762 | .872 | .359 | 2.120 |
|  | Dural injury | .338 | .714 | .358 | 1.423 |
|  | Anatomical repositioning of temporal muscle | .251 | .638 | .297 | 1.374 |
|  | Number of staples | .150 | .755 | .514 | 1.108 |
|  | Number of tiles | .451 | .941 | .802 | 1.103 |
|  | **Number of drains** | **.037** | **.536** | **.298** | **.964** |
|  | **Drain with suction** | **.021** | **.410** | **.193** | **.874** |
|  | Challenging skin closure | .801 | 1.128 | .443 | 2.874 |
|  | Scar crosses craniotomy | .305 | .668 | .310 | 1.443 |
|  | Intraoperative single shot antibiotics | .076 | .182 | .028 | 1.197 |
|  | Postoperative prophylactic antibiotic therapy | .438 | .713 | .304 | 1.674 |
|  | Blood loss | .198 | 1.001 | .999 | 1.003 |
|  | Intraoperative complications | .380 | .379 | .044 | 3.301 |
|  | Postoperative ICU stay | .377 | 1.361 | .687 | 2.697 |
|  | Postoperative cranial CT | .141 | 2.183 | .771 | 6.182 |
|  | **Any complication during hospitalization** | **<.001** | **6.243** | **2.798** | **13.929** |
|  | Postoperative ischemia | .389 | 3.002 | .246 | 36.700 |
|  | Postoperative CSF fistula | .872 | 1.214 | .115 | 12.811 |
|  | Postoperative seizure | .463 | 1.974 | .321 | 12.121 |
|  | New postoperative hydrocephalus | .999 | 8279918158 | .000 |  |
|  | Postoperative subdural hematoma | .287 | 2.064 | .543 | 7.841 |
|  | **Postoperative epidural hematoma** | **<.001** | **8.691** | **2.816** | **26.822** |
|  | Postoperative subgaleal hematoma | .096 | 3.102 | .819 | 11.749 |
|  | Postoperative meningitis | .999 | 9512680410 | .000 |  |
|  | Postoperative CSF leak | .179 | 4.189 | .519 | 33.849 |
|  | Postoperative wound infection | .998 | 1.107E+10 | .000 |  |
|  | Postoperative wound dehiscence | .999 | 7503262640 | .000 |  |
|  | Miscellaneous postoperative complication | .515 | 1.449 | .475 | 4.421 |
|  | CP performed before 1pm | .952 | .980 | .501 | 1.914 |
|  | **Postoperative length of stay** | **.004** | **1.077** | **1.024** | **1.133** |
|  | Time between DC and CP | .679 | 1.000 | .998 | 1.001 |
|  | **Early CP within 3 months of DC** | **.037** | **2.189** | **1.049** | **4.564** |
|  | Complications without reoperation | .064 | .411 | .160 | 1.052 |
|  | Resorption of autologous implant | .437 | 1.459 | .564 | 3.774 |
|  | Favorable long-term outcome | .421 | .740 | .356 | 1.539 |
| **Table 5** multivariate logistic regression model for independent variables and CPAR as the dependent variable, CP – cranioplasty, DC – decompressive craniectomy, OR – odds ratio, CI – confidence interval * represents a statistically significant value of p < 0.05 | | | | | |
